# Gamification of clinical decision‐making for medical physics residents

**DOI:** 10.1002/acm2.70907

**Published:** 2026-11-09

**Authors:** Katelyn Hasse, Alon Witztum, Dante Capaldi, Martina Descovich

**Affiliations:** ^1^ Department of Radiation Oncology University of California, San Francisco San Francisco California USA

**Keywords:** case‐based learning, clinical decision‐making, medical physics education

## Abstract

**Background:**

Clinical decision‐making is a cornerstone of effective clinical practice. For medical physics residency programs, clinical decision‐making can be a difficult skill to teach and comprehend. Most residents come from academic graduate programs with limited clinical exposure, and the regulatory nature of radiation oncology leads to limited opportunities for independent practice.

**Purpose:**

The purpose of this project was to incorporate clinical reasoning practice into a medical physics residency program.

**Methods:**

To this end, we have created a “Choose Your Own Adventure” case study‐based gamified lecture series. This series allows residents to have a safe space to practice making clinical decisions and explore potential outcomes. The residents are given an initial clinical scenario, three options of what could be done next, and asked to choose how they would proceed. The situation then evolves based on the choice that was made. After the “completion” of a scenario, there is a guided discussion, focusing on endpoints different choices might have led to. Finally, the group debriefs and walks through what might have happened in similar live clinical situations. Scenarios can be defined to reflect different aspects of the clinic and clinical decision‐making, and can have increasing difficulty to reflect different experience levels.

**Results:**

We have piloted a discussion series on clinical decision‐making in radiotherapy simulation and delivery for the past three years. This has been well received by both junior and senior medical physics residents, and has received positive evaluations in post‐session surveys completed by the residents. An example scenario is shared within the text, and a template of the presentation we use for this lecture series is shared in the Supplemental Material (Template S‐1).

**Conclusion:**

Overall, this lecture series is a practical teaching tool to support medical physics residency training by engaging trainees in clinical reasoning practice. We present an example scenario on Patient Safety. This concept can be applied to other medical physics clinical duties including Treatment Planning, Patient and Machine Quality Assurance, and Special Procedures.

## 1 INTRODUCTION

Clinical decision‐making, or clinical reasoning, is a cornerstone of effective education and a vital component of professional competence for clinicians.^1^ However, teaching this skill remains a significant challenge for medical physics residency programs. While the field is rooted in objective technical principles, actual clinical decisions are often subjective and context‐dependent. Translating theoretical knowledge into practice is further complicated by the limited duration of residencies, which often lack sufficient opportunities for supervised, progressive responsibility. For therapeutic medical physics residents, who often enter from academic programs with minimal clinical exposure, this mastery is especially difficult due to the highly regulated nature of radiation oncology, which inherently limits opportunities for independent practice.

Gamification is the process of using game elements and principles in non‐game contexts.^2^ In the context of education, gamification is the process of applying game elements to modify training content and methodology to help encourage interest and motivation in learning. Gamification can be used to help turn passive learning into interactive, hands‐on experiences. Key elements of effective gamification in medical education include Integration with Training Goals, Game Rules, Rapid Feedback, Fairness, Points or Scoring, Teamwork, Time Pressure, Increasing Difficulty, and Freedom to Fail.^3^

The gamification of clinical decision‐making builds on cognitivism and self‐determination theories, in that it provides opportunities for learners to practice critical thinking and problem‐solving skills, while simultaneously giving them autonomy, extrinsic motivation, and a sense of control over their learning.^4^, ^5^ Clinical reasoning is fundamentally a cognitive process that involves interpreting clinical cues and coming to a decision. Integrating autonomy‐supportive game mechanics, like meaningful choices, flexible pathways, enhance learner's engagement and cognitive growth.^6^

To this end, we have created a “Choose Your Own Adventure” case study‐based gamified lecture series as a way to integrate clinical decision‐making practice into our medical physics residency program. The “Choose Your Own Adventure” case study‐based lecture series format was derived from popular interactive gamebooks of the same name.^7^, ^8^ In these books, the reader is the protagonist who makes choices that determine the outcome of the book's plot. The outcomes could vary quite significantly depending on the reader's choices. This is also known as a ‘branched narrative″. This idea is consistent with Kolb's experiential learning theory, which suggests that hands‐on, engaging activities enhance learning and retention.^9^ This style has been used to enhance learning in other fields such as pharmacy^7^ and nursing^8^ but to our knowledge, this is the first application in the field of medical physics. The aim of this publication is to highlight the challenges of teaching clinical decision making to medical physics residents, and to provide a practical teaching tool to educators.

## 2 METHODS

The “Choose Your Own Adventure” lecture series allows learners to navigate through clinical decisions in an unfolding, interactive format, to allow residents to have a safe space to practice making decisions and explore potential outcomes. For each case, residents are given an initial clinical scenario with multiple possible initial decisions. The situation will then evolve based on the choice that was made, until the conclusion of the scenario has been reached. For our first lecture, scenarios focused on clinical physics coverage duties. The lecture series was developed in Microsoft Powerpoint, using slide hyperlinks to continue the narrative based on different decisions. A template for these slides, including one clinical example, can be found in the Supplemental Material (Template S‐1.pptx). The template was designed with the assistance of a large language model (ChatGPT Enterprise, Open AI), but the content was developed solely by the authors.

As shown in Table 1, the “Choose Your Own Adventure” series contains the top key elements of gamification in medical education including integration with training goals, game rules, rapid feedback, teamwork, time pressure, increasing difficulties and freedom to fail.^3^ These factors, combined with the competitive and social factors of the gamification, provide an engaging and effective learning experience.

**TABLE 1:** Key elements of gamification in medical education (Adapted from [3]).

Key Elements of Gamification in Medical Education (Adapted from [3])
Integration with educational objectives
Game Rules
Rapid Feedback
Points or Scoring
Teamwork
Competition
Time Pressure
Increasing Difficulty
Freedom to Fail

### 2.1 Game design

Each scenario starts with a descriptive blurb that puts the learner in the role of the physicist for a particular clinical situation. After the scenario is described, the learner reaches an initial decision point. The narrative then continues based on the initial decision. Subsequent decision points are designed to evaluate further aspects of resident knowledge, clinical reasoning skills and soft skills.

Scenarios can be designed to reflect different aspects of the clinic and clinical decision making that residents will likely experience as they become practicing physicists. They can be tailored to the resident experience level, with senior residents receiving more difficult scenarios to work through than junior residents.

Upon completion of each scenario, there is an opportunity to debrief and reflect, focusing on endpoints different choices might have led to. This allows for all learners to gain insight into the scenario. There is also an opportunity to walk through what might have happened in any live clinical scenarios, learn from any potential near‐misses or incidents, and follow up on any improvement to radiation processes and treatments that have since been implemented.

### 2.2 Game mechanics

As they traverse through the scenario, learners score one point for each decision point reached. They also receive extra points for flags. Flags might include decisions that could lead to patient safety events or not following through or taking responsibility for your tasks. Scoring was decided upon by consensus of four clinical physicists, and open to discussion with the residents. The learner with the lowest score after everyone has completed a scenario wins!

### 2.3 Resident perception

A post‐session survey was provided to the residents following the lecture. The survey had 6 questions, and was designed to evaluate their thoughts about the relevance and usefulness of the lecture. The survey can be seen below in Figure 1.

**FIGURE 1:**
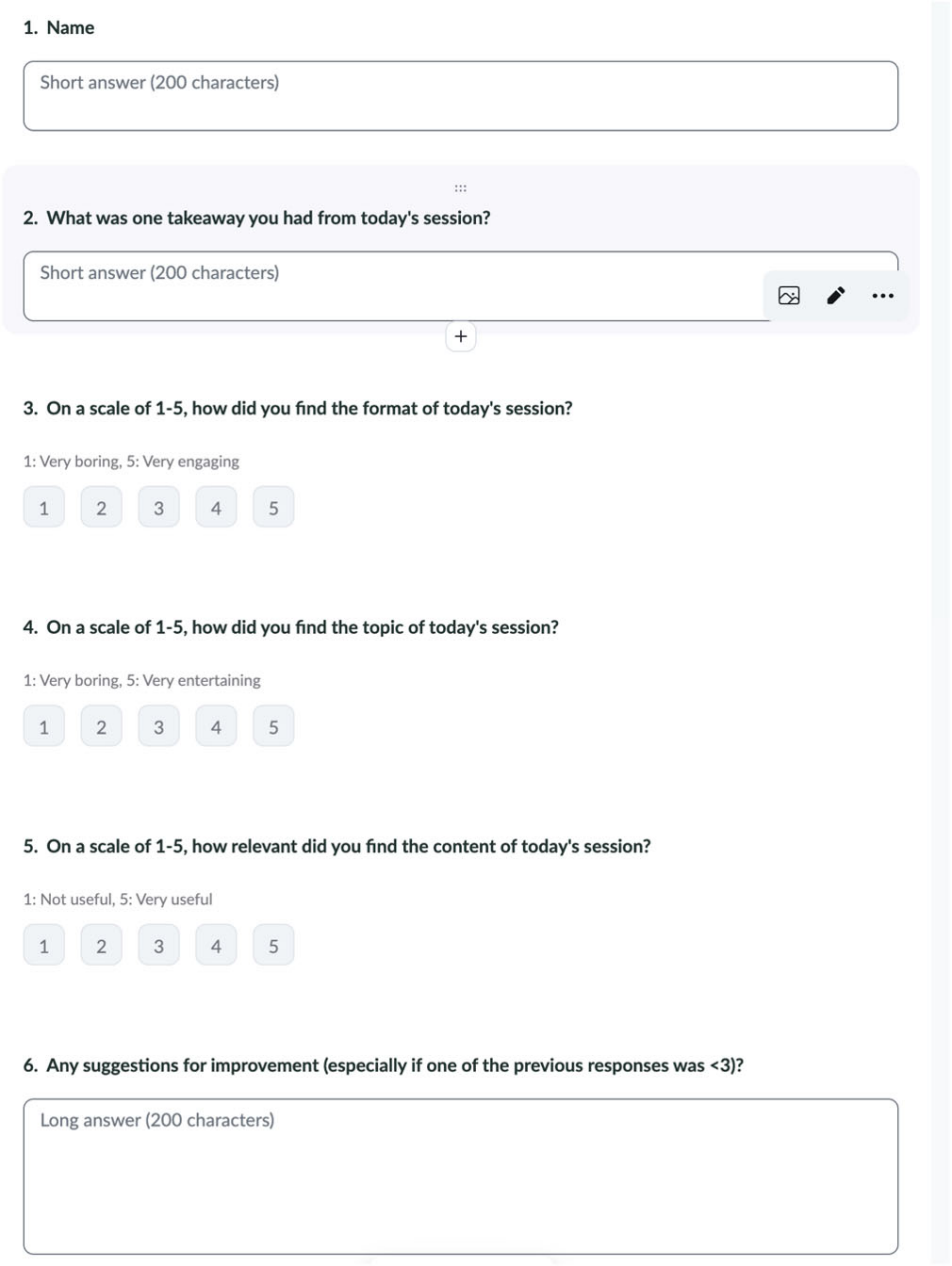
Survey given to medical physics residents upon completion of the “Choose‐Your‐Own‐Adventure” clinical physics duties case‐based lecture.

## 3 RESULTS

### 3.1 Game design

To illustrate the game design, we present an example scenario in Figure 2. In this example, the resident is called to the machine because the intercom has stopped working during patient treatment. The initial decision options are designed to test residents' knowledge of regulatory requirements for linac‐based radiation treatment,^10^ as well as survey resident knowledge of resources that are available for them within the clinical environment.

**FIGURE 2:**
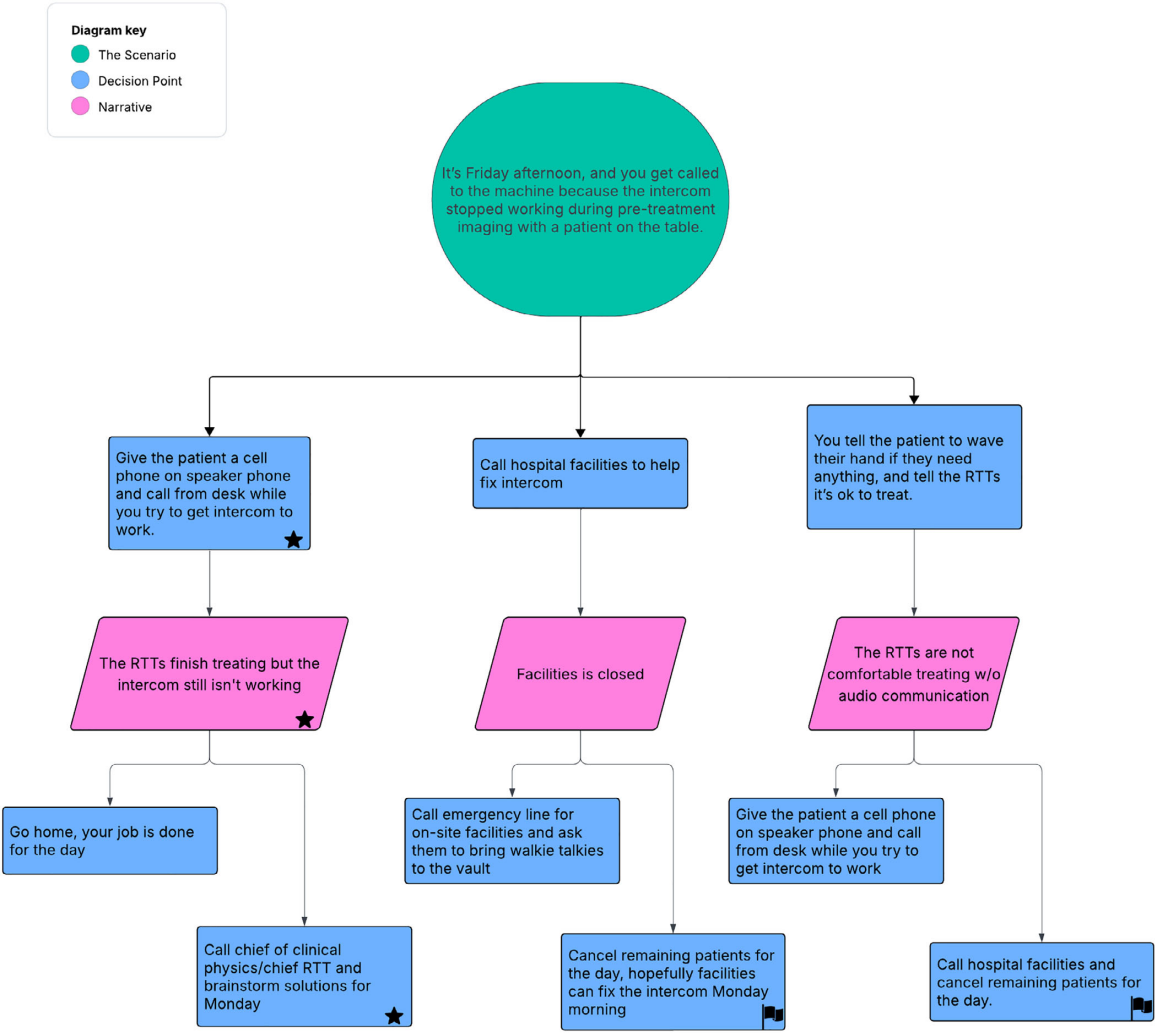
Example scenario for gamification of clinical decision‐making. The initial scenario is in green, decision points in blue, and narrative in pink. Stars indicate the fastest path through the scenario, and flags highlight less optimal decisions.

In Figure 2, the subsequent decisions rely on clinical reasoning skills and soft skills such as the ability to prioritize patient treatment, the ability to problem solve creatively, task ownership and follow through, and communication.

### 3.2 Game mechanism

The stars in Figure 2 illustrate the fastest path through the scenario. A resident choosing this scenario would receive a total of 3 points. Other paths might eventually reach the same conclusion, but the learner would have to go through more decision points. In practice for this scenario, this could lead to a treatment delay with the patient on the table. Flags for this scenario include not taking ownership or following through on tasks or canceling patient treatment so that you can go home for the day. If a resident were to choose the first two starred choices, but then decide to abdicate responsibility and go home, they would receive 5 points—an extra 2 points for the flag.

### 3.3 Resident perception

The “Choose Your Own Adventure” case‐based lecture series has been piloted with our medical physics residents over the past 3‐years since the development of this series (2023‐2026). The lecture series has been well‐received by both junior and senior medical physics residents, receiving a rating of 5 out of 5 for format, topic, and content (*n =* 4) in post session surveys. In particular, in annual review meetings, these lectures have also been called out as being the ‘most engaging’ of our didactic opportunities by junior residents, with the only suggestions for improvement being the expansion of our case library. Since the initial implementation, residents have also focused on highlighting clinical scenarios as future learning opportunities—noting situations such as recurring machine errors during treatment, daily QA device malfunction, software updates, patient setup discrepancies, and much more to be used as future cases in the lecture series.

## 4 DISCUSSION

Overall, the “Choose Your Own Adventure” case‐based lecture series is an interactive tool that can be used to engage medical physics residents in clinical reasoning practice. It allows an ethical and safe environment for exploring high‐stakes decisions in the Radiation Oncology clinical context.

### 4.1 Challenges, limitations, and future work

Challenges encountered during the development of the “Choose Your Own Adventure” case‐based lecture series include the labor‐intensive nature of developing comprehensive, multi‐decision scenarios within a realistic clinical context. The initial scenario development and templating took 3+ hours per scenario. We are investigating the possibility of using large language models, with output validated by clinicians, to assist with scenario development and branching narrative development. We are also investigating involving senior residents in scenario creation. Pedagogically, this would serve as a way for senior residents to reinforce and apply their learned clinical reasoning skills.

Limitations include the small sample size of learners and trainees that have provided feedback on the lecture series so far. We intend to continue developing the lecture series in a way that can be expanded to other institutions. Additionally, the evaluation of the impact of the Choose Your Own Adventure lecture series relies on learner's self‐reported perceptions versus objective skill gain. Educators are encouraged to look for other results as well, such as trainee participation and engagement in both the lecture series and in future clinical decision making.

In the future, we intend to expand the concept to create additional lectures focused on other clinical medical physics duties, including Treatment Planning, Chart Checks, Patient and Machine Quality Assurance, and Special Procedures. We also intend to include perspectives from the interprofessional clinical team. Scenarios (such as the example shown in Figure 1) often involve multiple team members, from radiation therapists to radiation oncology physicians and dosimetry. Their feedback and suggestions will add meaningful perspectives that will be extremely valuable to medical physics trainees and will help to instill the team dynamic present in the radiation oncology clinic.

## 5 CONCLUSION

The “Choose Your Own Adventure” case‐based lecture series is a practical teaching tool to support residents’ training by engaging them in clinical reasoning practice. It allows an ethical and safe environment for exploring high‐stakes decisions in the Radiation Oncology clinical context.

## AUTHOR CONTRIBUTIONS

K. Hasse—Conceptualization, methodology, visualization, writing—original draft. A. Witztum—Case development, implementation, software, writing—review and editing. D. Capaldi—Validation, survey implementation and analysis, writing—review and editing. M. Descovich—Educational framework development, methodology, case validation, writing—review and editing

## CONFLICT OF INTEREST STATEMENT

The authors declare no conflicts of interest.

## ETHICAL APPROVAL

This work describes the development and implementation of an educational activity designed to support clinical decision‐making training for medical physics residents. The activity was conducted as part of routine educational practice and did not involve patient interventions or the use of identifiable patient information. Clinical scenarios used in the educational materials were fictionalized or modified to prevent identification of individual patients. No identifiable information from participating learners is reported.

## Supporting information




Supporting Information

Supporting Information

Supporting Information

## Data Availability

The lecture slides presented in this Case Report are available in the supplemental information, and in other formats from the corresponding author upon reasonable request.
