# Supplementary material for: Gamification of clinical decision‐making for medical physics residents
Source: J Appl Clin Med Phys. 2026 Nov 9;27(11):e70907. doi: 10.1002/acm2.70907 (PMC13748818; doi:10.1002/acm2.70907)
Supplement: Supplementary file 1 [file ACM2-27-e70907-s001.pdf]

CHOOSE YOUR OWN ADVENTURE

Clinical Decision Pathways

A reusable interactive lecture template for medical physics residents

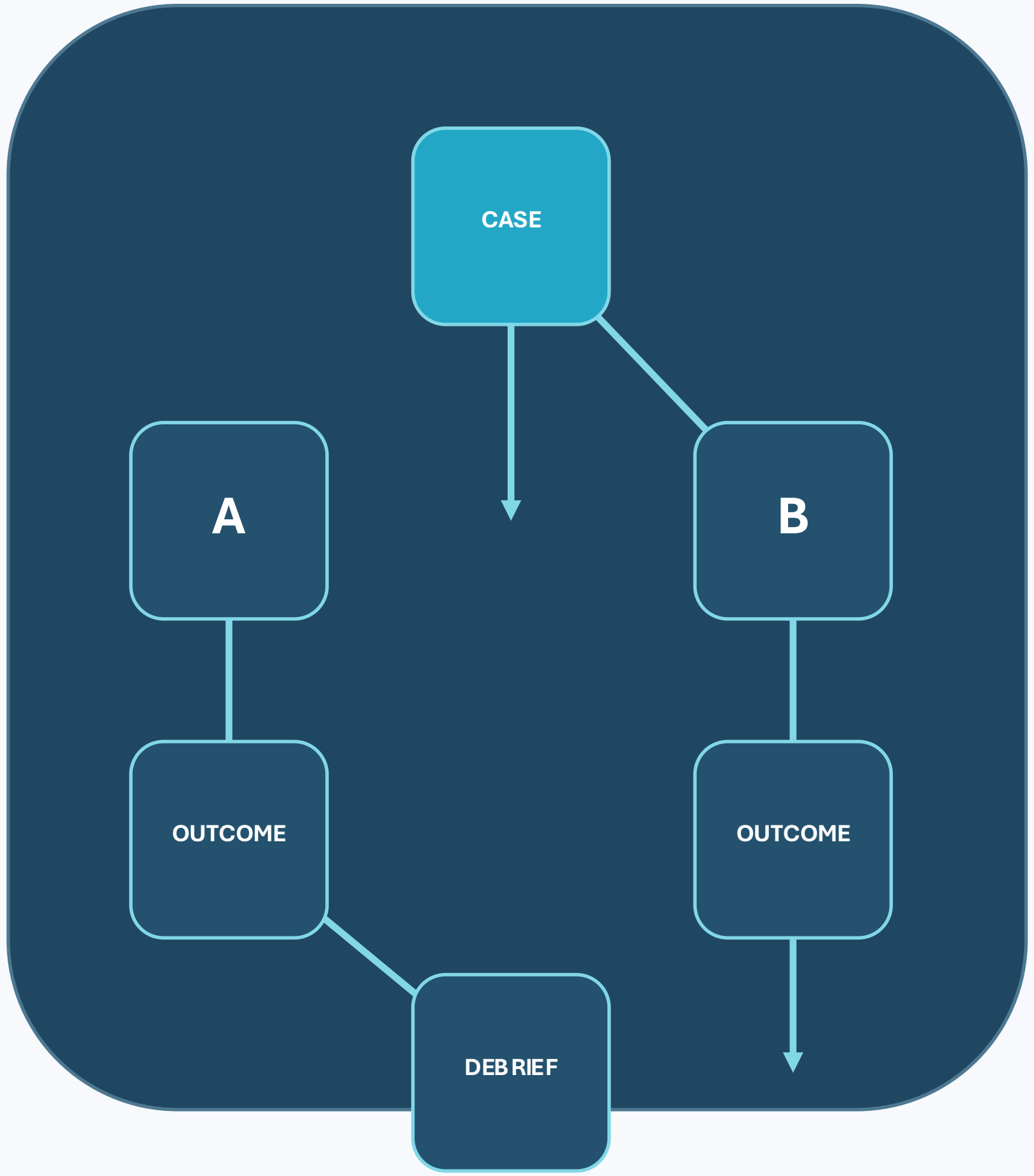

CLICK TO BEGIN

TEMPLATE

How this lecture works

Start with an example clinical scenario. Trainees will be asked to make a decision on how to proceed, and the narrative will branch from there.

1 Encounter

Present only the information available at that moment. Avoid telegraphing the “correct” physics answer.

2 Decide

Learners must choose an action, escalation path, measurement, or acceptance criterion.

3 Consequence

Reveal downstream clinical, dosimetric, workflow, and safety effects.

FACILITATOR RULE

Ask learners to understand the reasoning behind their decisions: What did you notice? What risk were you controlling? What would change your decision?

Learning objectives

Replace these with 2–4 decision-oriented objectives for each session.

1	**Recognize**	the physics information that is clinically relevant.
2	**Prioritize**	competing risks: accuracy, safety, urgency, resources, and patient impact.
3	**Escalate**	appropriately when uncertainty exceeds local tolerance or authority.
4	**Defend**	a decision using quantitative evidence and clinical context.

⌂ START ◇ MAP ← BACK

INSTRUCTOR VIEW

Branch map

Use this slide as a navigation hub. Duplicate branch modules as needed.

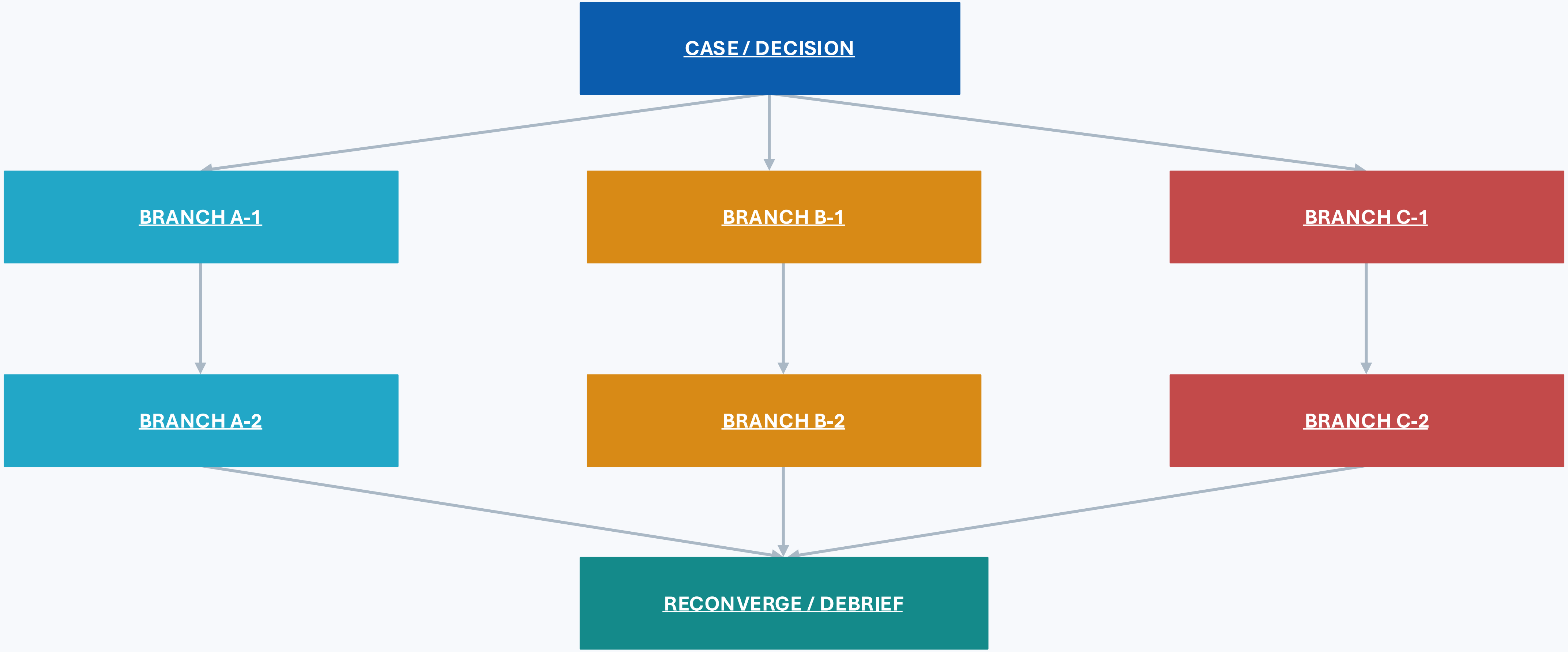

EXAMPLE DECISION SLIDE

Case 1: Can you hear me now?

Use this sample to demonstrate the interaction; replace with your own case.

What you know

- Friday afternoon
- Intercom has stopped working at linac
- Patient is on couch, has just completed pre-treatment imaging

What do you do next?

A **Tell therapists to resume treatment**

Tell the patient to wave their hands if they need anything

B **Pause treatment**

Call hospital facilities to see if they can help fix the intercom.

C **Get creative**

Give the patient a cell phone on speakerphone and call from the console..

⌂ START | ◇ MAP | ← BACK

BRANCH A

You tell the therapists to continue treatment, that the patient can wave their hands if they need anything.

New information

The radiation therapists are not comfortable treating without audio communication.

Discussion Points

- Should patients be asked to move during treatment?
- Are there any regulations related to audio or visual communication with patients during treatment?

What's next →

⌂ START ◇ MAP ← BACK

You pause treatment and call facilities

New information

Facilities is closed for the weekend.

Discussion points

What additional resources are available in a hospital setting?

Would this change at an academic vs community hospital?

What's next→

⌂ START ◇ MAP ← BACK

You get creative

Give the patient a cell phone on speakerphone and call from the console..

New information

The therapists are able to finish the Friday afternoon treatments, but the intercom still isn't working.

Discussion points

- Who should be involved in figuring out the next steps?
- What is the process between hand-offs at your institution?

What's next?→

⌂ START ◇ MAP ← BACK

Case 1A: Can you hear me now?

Use this sample to demonstrate the interaction; replace with your own case.

What you know

- Friday afternoon
- Intercom has stopped working at linac
- Patient is on couch, has just completed pre-treatment imaging
- RTTs are not comfortable treating without audio communication

What do you do next?

A **Get Creative**
Give the patient a cell phone on speakerphone and call from the console..

B **Pause treatment**
Call hospital facilities to see if they can help fix the intercom.

Continue to debrief →

⌂ START | ◇ MAP | ← BACK

Case 1B: Can you hear me now?

Use this sample to demonstrate the interaction; replace with your own case.

What you know

- Friday afternoon
- Intercom has stopped working at linac
- Patient is on couch, has just completed pre-treatment imaging
- Facilities is closed on Friday afternoon

What do you do next?

A **Pause treatment**

Call emergency line for on-site facilities and ask them to bring walki—talkies to the vault

B **Cancel treatment**

Cancel all remaining treatments, hopefully facilities can fix the intercom Monday morning.

Continue to debrief →

⌂ START | ◇ MAP | ← BACK

DECISION SLIDE

Case 1C: Can you hear me now?

Use this sample to demonstrate the interaction; replace with your own case.

What you know

- Friday afternoon
- Intercom has stopped working at linac
- Patient is on couch, has just completed pre-treatment imaging
- Facilities is closed on Friday afternoon
- The RTTs are able to finish patient treatments for the day but the intercom still isn't' working

What do you do next?

A **Team huddle**

Call chief of clinical physics and chief RTT to brainstorm solutions for Monday

B **Go home**

The patients have been treated, your job is done for the day

Continue to debrief →

⌂ START | ◇ MAP | ← BACK

DEBRIEF

Debrief the reasoning, not just the outcome

All branches return here. Capture the decision-making principles learners can transfer to the clinic.

01

What was the highest-risk failure mode?

02

Which piece of information changed your mind?

03

How would you escalate this situation were it to occur in the future?

04

What would you document—and why?

NEW CASE / NEXT DECISION →

⌂ START ◇ MAP ← BACK

Build each case around a real decision

A useful branch changes what the learner must weigh—not just which fact they recall.

CASE	Give only information available at decision time.
CHOICES	Use plausible actions a resident might actually consider.
CONSEQUENCE	Show tradeoffs; avoid “gotcha” punishment.
TEACH	Name the physics principle, uncertainty, and local workflow.
RECONVERGE	Extract a transferable decision rule.

⌂ START ◇ MAP ← BACK

DUPLICATE ME

Blank decision slide

Replace bracketed text, then link each choice to its branch slide.

[CASE SNAPSHOT]

- [Clinical context]
- [Relevant imaging / measurement]
- [Constraint or uncertainty]
- [What the resident knows now]
- [What they do NOT know yet]

[DECISION QUESTION?]

A **[Choice A]**
[Short rationale / action]

B **[Choice B]**
[Short rationale / action]

C **[Choice C]**
[Short rationale / action]

⌂ START ◇ MAP ← BACK
