# Supplementary material for: Gamification of clinical decision‐making for medical physics residents
Source: J Appl Clin Med Phys. 2026 Nov 9;27(11):e70907. doi: 10.1002/acm2.70907 (PMC13748818; doi:10.1002/acm2.70907)
Supplement: Supplementary file 2 [file ACM2-27-e70907-s002.docx]

**Gamification of Clinical Decision-Making for Medical Physics Residents**

Katelyn Hasse, Alon Witztum, Dante Capaldi, Martina Descovich

Department of Radiation Oncology, University of California, San Francisco

San Francisco, CA

Corresponding Author: [katelyn.hasse@ucsf.edu](mailto:katelyn.hasse@ucsf.edu)

Funding Statement: *The authors declare that no direct funding or external financial support was received for the research, authorship, or publication of this article.*

Data Availability Statement: *The lecture slides presented in this Case Report are available in the supplemental information, and in other formats from the corresponding author upon reasonable request.*

Conflict of Interest Statement: *The authors have no conflicts to disclose relevant to this article.*

Author Contribution Statement:

- K. Hasse – Conceptualization, methodology, visualization, writing – original draft
- A. Witztum – Case development, implementation, software, writing – review and editing
- D. Capaldi – Validation, survey implementation and analysis, writing – review and editing
- M. Descovich – Educational framework development, methodology, case validation, writing – review and editing

Ethics Statement: *This work describes the development and implementation of an educational activity designed to support clinical decision-making training for medical physics residents. The activity was conducted as part of routine educational practice and did not involve patient interventions or the use of identifiable patient information. Clinical scenarios used in the educational materials were fictionalized or modified to prevent identification of individual patients. No identifiable information from participating learners is reported.*
